# Clinical Utility of a Nomogram for Predicting 30-Days Poor Outcome in Hospitalized Patients With COVID-19: Multicenter External Validation and Decision Curve Analysis

**DOI:** 10.3389/fmed.2020.590460

**Published:** 2020-12-23

**Authors:** Bin Zhang, Qin Liu, Xiao Zhang, Shuyi Liu, Weiqi Chen, Jingjing You, Qiuying Chen, Minmin Li, Zhuozhi Chen, Luyan Chen, Lv Chen, Yuhao Dong, Qingsi Zeng, Shuixing Zhang

**Affiliations:** ^1^Department of Radiology, The First Affiliated Hospital of Jinan University, Guangzhou, China; ^2^Department of Radiology, The First Affiliated Hospital of Guangzhou Medical University, Guangzhou, China; ^3^Zhuhai Precision Medical Center, Zhuhai People's Hospital (Zhuhai Hospital Affiliated With Jinan University), Zhuhai, China; ^4^Big Data Decision Institute, Jinan University, Guangzhou, China; ^5^Guangdong Provincial People's Hospital, Guangdong Academy of Medical Sciences, Guangzhou, China

**Keywords:** nomogram, poor outcome, COVID-19, CT score, clinical usefulness

## Abstract

**Aim:** Early detection of coronavirus disease 2019 (COVID-19) patients who are likely to develop worse outcomes is of great importance, which may help select patients at risk of rapid deterioration who should require high-level monitoring and more aggressive treatment. We aimed to develop and validate a nomogram for predicting 30-days poor outcome of patients with COVID-19.

**Methods:** The prediction model was developed in a primary cohort consisting of 233 patients with laboratory-confirmed COVID-19, and data were collected from January 3 to March 20, 2020. We identified and integrated significant prognostic factors for 30-days poor outcome to construct a nomogram. The model was subjected to internal validation and to external validation with two separate cohorts of 110 and 118 cases, respectively. The performance of the nomogram was assessed with respect to its predictive accuracy, discriminative ability, and clinical usefulness.

**Results:** In the primary cohort, the mean age of patients was 55.4 years and 129 (55.4%) were male. Prognostic factors contained in the clinical nomogram were age, lactic dehydrogenase, aspartate aminotransferase, prothrombin time, serum creatinine, serum sodium, fasting blood glucose, and D-dimer. The model was externally validated in two cohorts achieving an AUC of 0.946 and 0.878, sensitivity of 100 and 79%, and specificity of 76.5 and 83.8%, respectively. Although adding CT score to the clinical nomogram (clinical-CT nomogram) did not yield better predictive performance, decision curve analysis showed that the clinical-CT nomogram provided better clinical utility than the clinical nomogram.

**Conclusions:** We established and validated a nomogram that can provide an individual prediction of 30-days poor outcome for COVID-19 patients. This practical prognostic model may help clinicians in decision making and reduce mortality.

## Introduction

The rapid spread of coronavirus disease 2019 (COVID-19) caused by severe acute respiratory syndrome coronavirus 2 (SARS-CoV-2) as a potentially fatal disease is a major and urgent threat to global health (1). As of July 9, 2020, there are more than 12.14 million confirmed cases by the World Health Organization (WHO) with 551,044 deaths (2). The clinical spectrum of COVID-19 ranges from mild to critically ill. Most COVID-19 patients had mild acute respiratory infection symptoms, such as fever, dry cough, and fatigue, but some could rapidly develop fatal complications, including respiratory failure, multiple organ dysfunction, shock, or even death (3). To date, no specific treatments were recommended for COVID-19 except for meticulous supportive care (4); therefore, early identification of patients with poor prognosis may facilitate the provision of proper supportive treatment in advance and reduce mortality.

The profusion of data requires machine learning to improve and accelerate COVID-19 diagnosis, treatment, and prognosis (5). Machine learning uses patterns in data to improve performance or make accurate predictions (6). It provides a powerful set of tools to unravel the relationship between the variables and outcomes, particularly when data are non-linear and complex (7). At present, some early warning models using machine learning for predicting COVID-19 patients at risk of developing a severe or critical condition have been reported (8–14). Such models are usually assessed with statistical measures for discrimination and calibration. Theoretically, a model with better discrimination and calibration indicates a better guide to clinical management, whereas statistical measures fall short when we want to determine whether the risk model improves clinical decision-making (15). Such measures cannot inform us whether it is beneficial to apply a model to make clinical decisions or which of two models lead to better decisions, especially if they have similar discrimination and calibration. As compared to traditional performance metrics, decision curve analysis (DCA) can assess the clinical utility of models for decision-making (16). DCA plots net benefit at a range of clinically reasonable risk thresholds (16). It identifies risk models that can help us make better clinical decisions.

Therefore, the purpose of this study was to develop and validate a prognostic machine-learning model based on clinical, laboratory, and radiological features of COVID-19 patients at hospital admission for 30-days poor outcome assessment during hospitalization. We also used the DCA and clinical impact curve (CIC) analysis to evaluate the clinical utility and net benefit of the predictive model in supporting clinical decisions. This model may serve as a tool for early identification of COVID-19 patients at high risk for poor outcomes during hospitalization.

## Materials and Methods

### Patients

This study was approved by the institutional review board and the need for written informed consent was waived. A total of 233 confirmed COVID-19 from two designated hospitals of Wuhan, Hubei province of China were consecutively and retrospectively included between January 3 to March 20, 2020. The inclusion criteria were as follows: (1) patients with a laboratory-confirmed COVID-19, which was achieved by real-time reverse transcription-polymerase chain reaction (RT-PCR) assay of throat swab samples (at least two samples were taken, at least 24 h apart) for COVID-19 according to the protocol established by the WHO; and (2) patients received treatment at hospitals. The exclusion criteria were as follows: (1) patients with critical diseases at presentation. This exclusion allows unbiased analysis, for predicting deterioration in patients during their hospitalization; (2) time interval more than 2 days from admission to examinations because delayed testing may skew the inclusion set to a more critical status; and (3) unavailable clinical and laboratory data. The primary cohort was randomly divided into two datasets, 80% for training, and the remaining 20% for internal validation using 5-fold cross-validation. Two externally validation cohorts were under the same inclusion and exclusion criteria. The external validation cohorts included patients hospitalized for COVID-19 between January 3 and May 21, 2020. Finally, 110 and 118 COVID-19 patients were enrolled in the external validation cohort 1 (Tianmen, Hubei province) and cohort 2 (Dongguan, outside Hubei province), respectively.

### Data Collection

After consultation with physicians in charge of COVID-19 patients and review of the recent literature regarding the prognosis of COVID-19 on the dataset of PubMed using the terms “COVID,” “SARS-CoV-2,” “prognosis,” “poor outcome,” “severe,” and “critically ill,” a set of clinical, laboratory, and radiological characteristics were identified and the data were collected from the electronic medical records. The clinical characteristics included demographics, comorbidities, and symptoms. Laboratory parameters were recorded, including complete blood count, D-dimer, C-reactive protein (CRP), cardiac enzymes, procalcitonin, liver function test, kidney function test, fasting blood glucose (FBG), and electrolyte. The data in source documents were confirmed independently by at least two researchers. We also calculated the neutrophil–lymphocyte count ratio as it is an important risk factor of disease severity. Imputation for missing variables was considered if missing values were <15%. We used mean value to impute numeric features.

A semiquantitative CT scoring system was designed to assess the involvement degree or area of pneumonia for every single lobe (total five lung lobes): 0 for 0% involvement; 1 for 1–25% involvement; 2 for 26–50% involvement; 3 for 51–75% involvement; 4 for 76–100% involvement. The final CT score (range, 0–20) was assigned by summarizing the total scores of five lobes (17). CT images were reviewed independently by two radiologists with more than 10 years of experience, who were blinded to clinical and laboratory results. Any discrepancy was resolved by a consensus viewing. The detailed CT acquisition and reconstruction parameters are presented in the Supplementary Document.

### Predictive Variable Selection and Clinical Score Development

Clinical variable selection and risk score development were only performed on the primary cohort as an independent process and ultimately evaluated by the external validation cohort 1 and 2. Pearson correction (PCC) analysis was first used to assess the correlation between variable pairs; a PCC of 0.9 was usually used to eliminate the redundancy in previous studies (18–20). However, most of the variables remained relatively independent in our work (PCC <0.9), with only one pair of variables' (serum creatinine and procalcitonin) coefficients exceeding 0.86. Considering the significance of PCC analysis for variable selection and modeling, we set the cutoff value to 0.86. If the PCC value of the variable pair was larger than 0.86, we calculated the correlation between these two variables and the label and removed one of the slightly unrelated variables. After this process, the dimension of the variable space was reduced, and each variable was independent of each other. Then, the least absolute shrinkage and selection operator (LASSO) logistic regression algorithm was used for further variable selection and development of the clinical score for the 30-days poor outcome prediction. The complexity and performance of the LASSO algorithm relies crucially on the choice of the tuning parameter λ, and the larger λ penalizes the linear model more, resulting in a model with fewer variables. The predictors with non-zero LASSO coefficients based on the 1 standard error rule and penalty parameter tuning were identified using 5-fold cross-validation and the minimum criteria of the area under the receiver-operator characteristic (ROC) curve (AUC). Afterward, the selected clinical variables were combined linearly to construct a clinical score.

### Clinical and Clinical-CT Nomogram Construction and Validation

For the superior clinical variables, we conducted the clinical nomogram to visualize the relationship between the variables and predicted probabilities. In addition, a multivariable logistic regression analysis was applied to integrate the predictive clinical variables with the CT score to construct a clinical-CT nomogram. By applying 5-fold cross-validation on the primary cohort, the performance of two constructed nomograms was first internally validated using the AUC metric and then externally validated by the external validation cohort 1 and 2. The clinical score and clinical-CT score for each patient were computed, and the association between the score and COVID-19 30-days poor outcome was assessed with the Mann–Whitney *U*-test. Subsequently, the calibration properties reflecting goodness of fit of the nomograms generated were assessed by plotting the predicted probabilities against the observed event proportions, subjected to bootstrapping validation (1,000 bootstrap resamples) and the Hosmer–Lemeshow test. The degree of overlap between the calibration curve and the diagonal reflects the predictive accuracy of the proposed nomograms.

### Clinical Usefulness of the CT Score, Clinical Nomogram, and Clinical-CT Nomogram

DCA can be used to estimate the net benefit of a model based on the difference between the number of true-positive and false-positive results, weighted by the odds of the selected threshold probability of risk (21). To assess the clinical usefulness of the predictive models, the decision curves for each model were then compared with those for the two default strategies where patients are managed without the use of a model: “treating all” or “treating none” (22). The “treat all” strategy assumes that doctors will treat all patients regardless of their risk estimates. The “treat none” assumes that all patients are at low risk that none of them is treated. The net benefit is dependent on the threshold probability that defines “high risk” of critical illness. A predictive model has clinical utility if its net benefit curve is above that of “treat all” or “treat none” for a range of reasonable risk thresholds. The model with higher net benefit for a certain risk or probability has more clinical utility. The clinicians could refer to this to determine whether clinical decision-making based on the models will do better than harm. On this basis, we further plotted the CIC of the models. The CIC shows the estimated number who would be declared high risk for each risk threshold and visually showed the proportion of those who were cases (true positives).

### Definition of Clinical Endpoint

We defined the severity of COVID-19 according to the newest COVID-19 guidelines released by the National Health Commission of China (23) and the guidelines of the American Thoracic Society for community-acquired pneumonia (24). Thirty-day poor outcome is defined as meeting at least one of the following criteria within 30 days after admission to hospital: respiratory failure requiring mechanical ventilation, shock, intensive care unit (ICU) admission, multiple organ dysfunction, or death.

### Statistical Analysis

Categorical variables were expressed as counts and percentages, while continuous variables are shown as median and interquartile range. Continuous variables were compared using the Mann–Whitney *U*-test, and categorical variables were compared using the chi-square test. PCC analysis, variable selection, clinical score development, and nomogram construction were conducted with R statistical software version 3.5.1 (http://www.R-project.org). The values difference of each selected superior variable within COVID-19 risk groups was compared by the Mann–Whitney *U*-test. Similarly, the test was applied to the comparison of clinical score and clinical-CT score between low-risk and high-risk groups. The performance of the CT score and nomograms were evaluated by AUC, sensitivity, specificity, accuracy, positive predictive value (PPV), as well as negative predictive value (NPV). ROC curves of the two nomograms were compared by the method of DeLong et al. using the MedCalc version 15.2.2 (MedCalc Inc., Mariakerke, Belgium). Note that a two-tailed *p* < 0.05 indicated statistical significance. All the cutoff values of ROC curves were determined by the principle of the maximum Youden index within the primary cohort. Patients were stratified into low-risk or high-risk group according to the cutoff value. We reported our findings in accordance with the Guidelines for Standards for Reporting Diagnostic accuracy studies, Developing and Reporting Machine Learning Predictive Models in Biomedical Research, and Transparent Reporting of a Multivariable Prediction Model for Individual Prognosis or Diagnosis.

## Results

### Demographic and Clinical Characteristics

Of 233 patients hospitalized for COVID-19 in the primary cohort, the mean age was 55.4 years ± 16.9 (interquartile range, 42–67 years), and 129 (55.4%) were male. Of 228 patients hospitalized for COVID-19 in two cohorts at test datasets, the mean age was 46.2 years ± 17.7 (interquartile range, 35–58 years) and 135 (59.2%) were male. The incidence of 30-days poor outcome in the primary cohort, external validation cohorts 1 and 2 was 20.2, 10.9, and 16.1%, respectively. Clinical characteristics and CT scores of the primary cohort and external validation cohorts 1 and 2 are summarized in Table 1 and Supplementary Table 1, respectively.

**Table 1:** Clinical and laboratory characteristics among patients in the primary cohort with or without poor outcome.

	**Total (*n* = 233)**	**30-days poor outcome**		
		**Yes (*n* = 47)**	**No (*n* = 186)**	***P*-value**
**Age (years)**, ***n*** **(%)**	58.0 (42.0, 67.0)	67.0 (60.0, 78.0)	54.5 (40.8, 65.0)	<0.001
**Sex**, ***n*** **(%)**				
Male	129 (55.4)	32 (68.1)	97 (52.2)	0.050
Female	104 (44.6)	15 (31.9)	89 (47.8)	
**Comorbidities**				
Hypertension	53 (22.7)	18 (38.3)	35 (18.8)	0.004
Coronary heart diseases	19 (8.2)	7 (14.9)	12 (6.5)	0.073
Diabetes	28 (12.0)	12 (25.5)	16 (8.6)	0.001
Hepatitis	8 (3.4)	0	8 (4.3)	0.364
Chronic lung diseases	20 (8.6)	4 (8.5)	16 (8.6)	1.000
**Symptoms and signs**				
Fever	142 (60.9)	40 (85.1)	102 (54.8)	<0.001
Cough	93 (39.9)	23 (48.9)	70 (37.6)	0.157
Sputum	25 (10.7)	8 (17.0)	17 (9.1)	0.119
Headache	1 (0.4)	1 (2.1)	0 (0.0)	0.202
Sore throat	5 (2.1)	2 (4.3)	3 (1.6)	0.265
Fatigue	17 (7.3)	3 (6.4)	14 (7.5)	1.000
Myalgia	1 (0.4)	0	1 (0.5)	1.000
Chest pain/Chest distress	13 (5.6)	1 (2.1)	12 (6.5)	0.475
Shortness of breath	26 (11.2)	11 (23.4)	15 (8.1)	0.003
Diarrhea	4 (1.7)	2 (4.3)	2 (1.1)	0.182
Chills	2 (0.9)	1 (2.1)	1 (0.5)	0.363
Asymptomatic	52 (22.3)	0	52 (28.0)	<0.001
**Laboratory findings, median (IQR)**				
WBC (× 10^9^/L)	5.3 (4.1, 6.7)	6.0 (4.4, 9.4)	5.1 (4.1, 6.4)	0.011
Neutrophil (× 10^9^/L)	3.5 (2.6, 5.1)	5.1 (3.2, 8.7)	3.2 (2.5, 4.3)	<0.001
Lymphocyte (× 10^9^/L)	1.1 (0.7, 1.6)	0.7 (0.4, 1.0)	1.2 (0.9, 1.7)	<0.001
NLR	3.0 (1.8, 6.0)	8.0 (3.3, 14.8)	2.6 (1.7, 4.3)	<0.001
LDH (U/L)	204.0 (154.0, 287.5)	394.3 (277.0, 550.0)	185.0 (147.0, 244.8)	<0.001
Hemoglobin (g/L)	130.0 (119.5, 143.5)	134.0 (123.0, 146.0)	128.0 (119.0, 142.0)	0.217
Platelet (g/L)	200.0 (148.5, 252.2)	165.0 (125.0, 222.0)	210.5 (153.5, 260.3)	0.007
Albumin (g/L)	37.3 (32.4, 41.0)	31.9 (29.2, 35.5)	38.4 (34.5, 42.0)	<0.001
AST (U/L)	23.0 (16.5, 36.0)	38.0 (27.0, 58.0)	20.0 (15.0, 31.5)	<0.001
ALT (U/L)	22.0 (15.0, 36.5)	26.0 (18.0, 41.0)	21.0 (15.0, 35.0)	0.061
DBIL (μmol/L)	3.6 (2.6, 5.0)	4.2 (3.2, 6.9)	3.4 (2.5, 4.5)	0.002
IBIL (μmol/L)	7.3 (5.2, 10.2)	5.7 (4.5, 7.6)	7.8 (5.6, 10.8)	0.001
TBIL (μmol/L)	11.0 (8.3, 15.1)	9.8 (8.1, 14.6)	11.3 (8.4, 15.1)	0.444
APTT (s)	34.2 (31.8, 36.9)	35.3 (31.1, 38.4)	34.1 (32.0, 36.5)	0.236
PT (s)	13.3 (12.5, 14.3)	14.0 (13.0, 15.0)	13.2 (12.5, 14.0)	0.001
D-dimer (μg/ml)	0.3 (0.1, 0.9)	0.8 (0.3, 8.0)	0.2 (0.1, 6.3)	<0.001
Serum creatinine (μmol/L)	70.0 (58.0, 82.0)	79.0 (67.0, 99.0)	68.0 (57.0, 80.0)	<0.001
hs-CRP (mg/L)	12.2 (1.3, 36.0)	36.5 (32.2, 38.2)	3.7 (0.8, 27.2)	<0.001
Procalcitonin (ng/ml)	0.2 (0.1, 2.9)	0.5 (0.2, 4.9)	0.2 (0.1, 1.2)	<0.001
Potassium (mmol/L)	4.0 (3.7, 4.4)	3.9 (3.5, 4.5)	4.0 (3.7, 4.4)	0.174
Sodium (mmol/L)	139.0 (138.0, 141.0)	138.0 (134.0, 140.0)	140.0 (138.5, 142.0)	<0.001
Chloride (mmol/L)	105.0 (103.0, 107.0)	103.0 (99.0, 107.0)	106.0 (104.0, 107.0)	<0.001
FBG (mmol/L)	5.6 (4.8, 7.0)	7.6 (6.3, 11.9)	5.3 (4.8, 6.3)	<0.001

*Data were median (interquartile range, IQR) or number (percentage). P-values were calculated by Mann–Whitney U-test, χ^2^ test, or Fisher's exact test, as appropriate*.

*WBC, white blood cells; LDH, lactate dehydrogenase; AST, aspartate aminotransferase; ALT, alanine aminotransferase; TBIL, total bilirubin; DBIL, direct bilirubin; IBIL, indirect bilirubin; APTT, activated partial thromboplastin time; PT, prothrombin time; hs-CRP, high-sensitivity C-reactive protein; NLR, neutrophil–lymphocyte ratio; FBG, fasting blood glucose*.

### Variable Selection and Clinical Score Development

**Figure 1:**
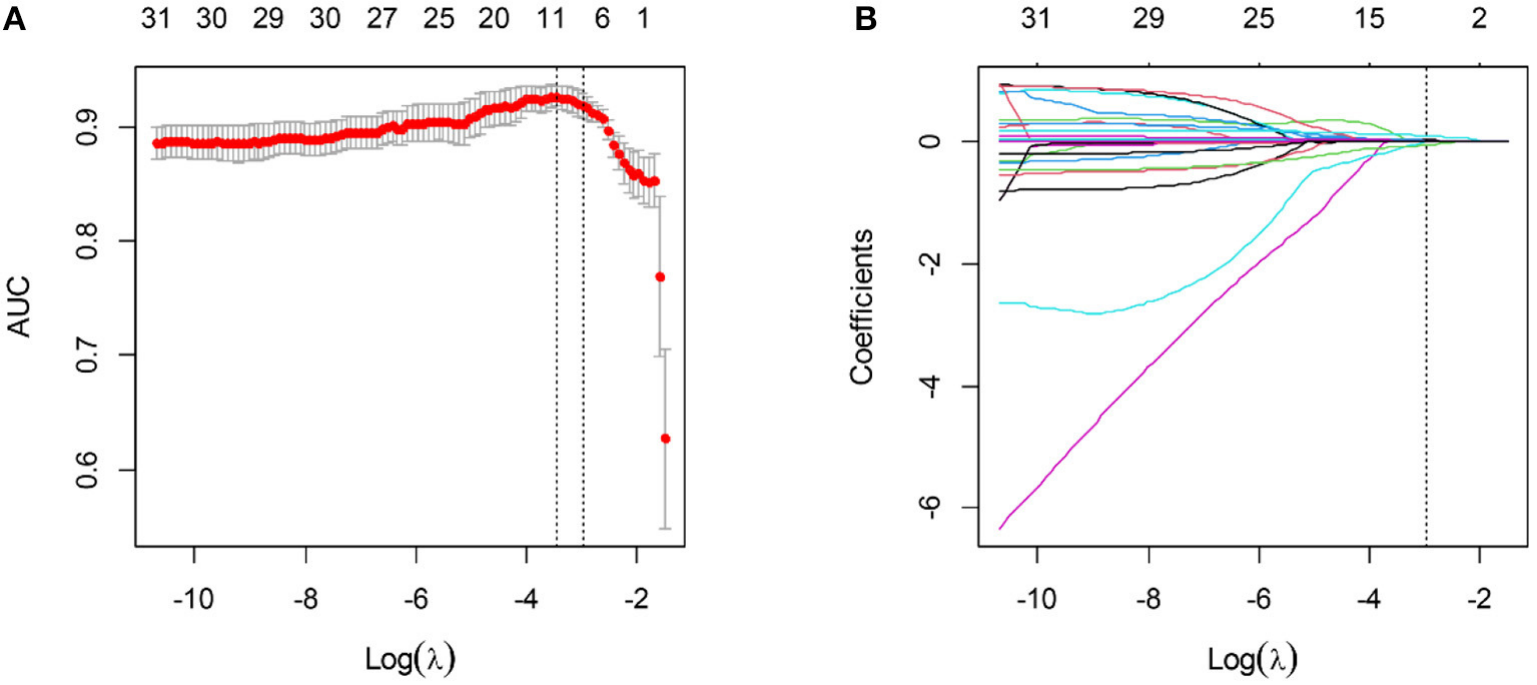
Variable selection using the LASSO algorithm. **(A)** The optimal tuning parameter λ was selected based on the minimum criteria of AUC using 5-fold cross-validation. λ adjusts the regularization penalty to control the complexity of the clinical score, and the optimal value was 0.051. **(B)** LASSO coefficient profiles of the variables. The vertical line was drawn at the selected log (λ), where eight clinical variables with non-zero coefficients were selected.

**Figure 2:**
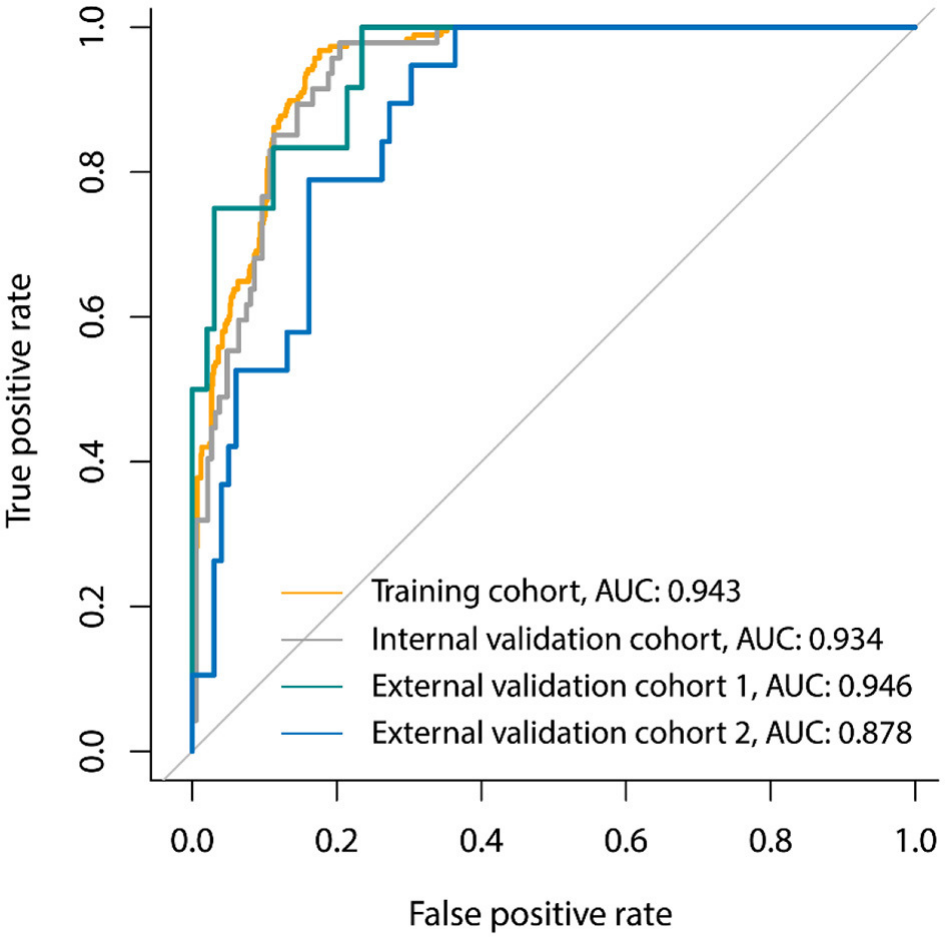
Receiver-operator characteristic (ROC) curves of the clinical signature for 30-days poor outcome prediction in the primary cohort, internal validation cohort, and two external validation cohorts.

Three laboratory parameters uric acid, creatine kinase, and creatine kinase MB with missing value >15% were excluded for the variable selection. Most of the variables remained relatively independent in this study (PCC <0.9), with only one pair of variable coefficients exceeding 0.86 (serum creatinine and procalcitonin). Thus, procalcitonin with low correlation with 30-days poor outcome in hospitalized patients with COVID-19 was removed after PCC analysis, and the remaining 31 variables were initially selected. Then, eight superior variables with non-zero coefficients were identified across 5-fold cross-validation using the LASSO regression, with the optimization tuning parameter of 0.051 (Figure 1). Supplementary Figure 1 depicts the value distribution of the identified eight superior clinical variables in patients with good or poor 30-days outcome across all the cohorts. In Figure 2, the clinical score constructed by linearly integrating the eight clinical variables achieved an AUC of 0.943 (95% CI: 0.927–0.957), sensitivity of 96.8%, specificity of 82.4%, accuracy of 85.2%, PPV of 58.0%, and NPV of 99.0% in the training cohort and an AUC of 0.934 (95% CI: 0.894–0.963), sensitivity of 93.6%, specificity of 80.7%, accuracy of 83.3%, PPV of 55.0%, and NPV of 98.0% in the internal validation cohort. When tested, the clinical score yielded excellent performance in the external validation cohort 1 (AUC, 0.946, 95% CI: 0.886–0.980; sensitivity, 100%; specificity, 76.5%; accuracy, 79.1%; PPV, 34.3%; and NPV, 100%), and external validation cohort 2 (AUC, 0.878, 95% CI: 0.805–0.931; sensitivity, 79.0%; specificity, 83.8%; accuracy, 82.2%; PPV, 46.9%; and NPV, 95.3%). The patients could be stratified into low-risk or high-risk group according to the optimal cutoff value of 0.190.

### Predictive Performance of the Clinical and Clinical-CT Nomogram

**Figure 3:**
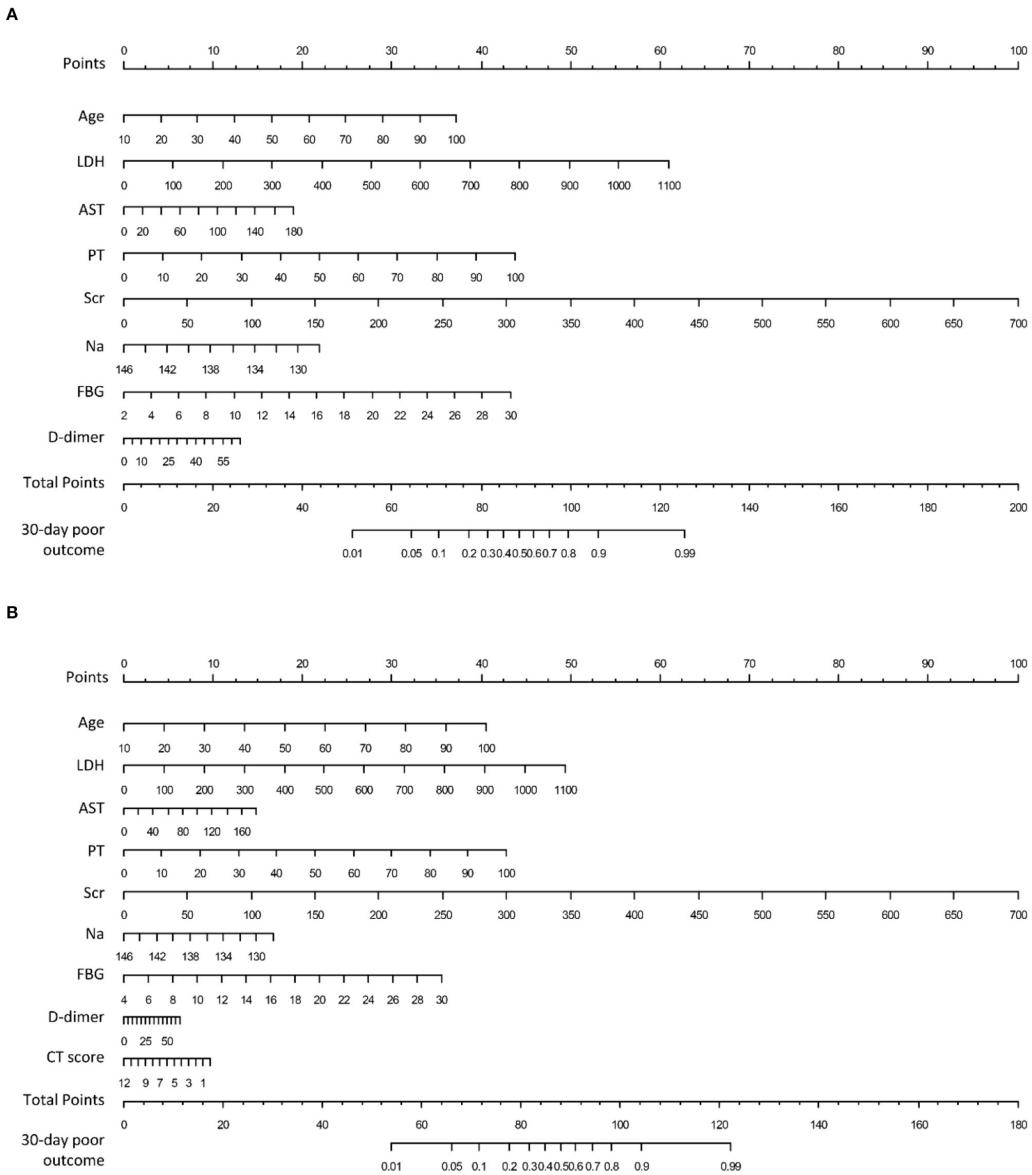
Nomograms to predict 30-days poor outcome in patients hospitalized for COVID-19. **(A)** Clinical nomogram constructed by linearly integrating the eight clinical variables; **(B)** clinical-CT nomogram constructed by linearly integrating the eight clinical variables and CT score. LDH, lactic dehydrogenase; AST, aspartate aminotransferase, PT, prothrombin time; Scr, serum creatinine; Na, serum sodium; FBG, fasting blood glucose.

**Figure 4:**
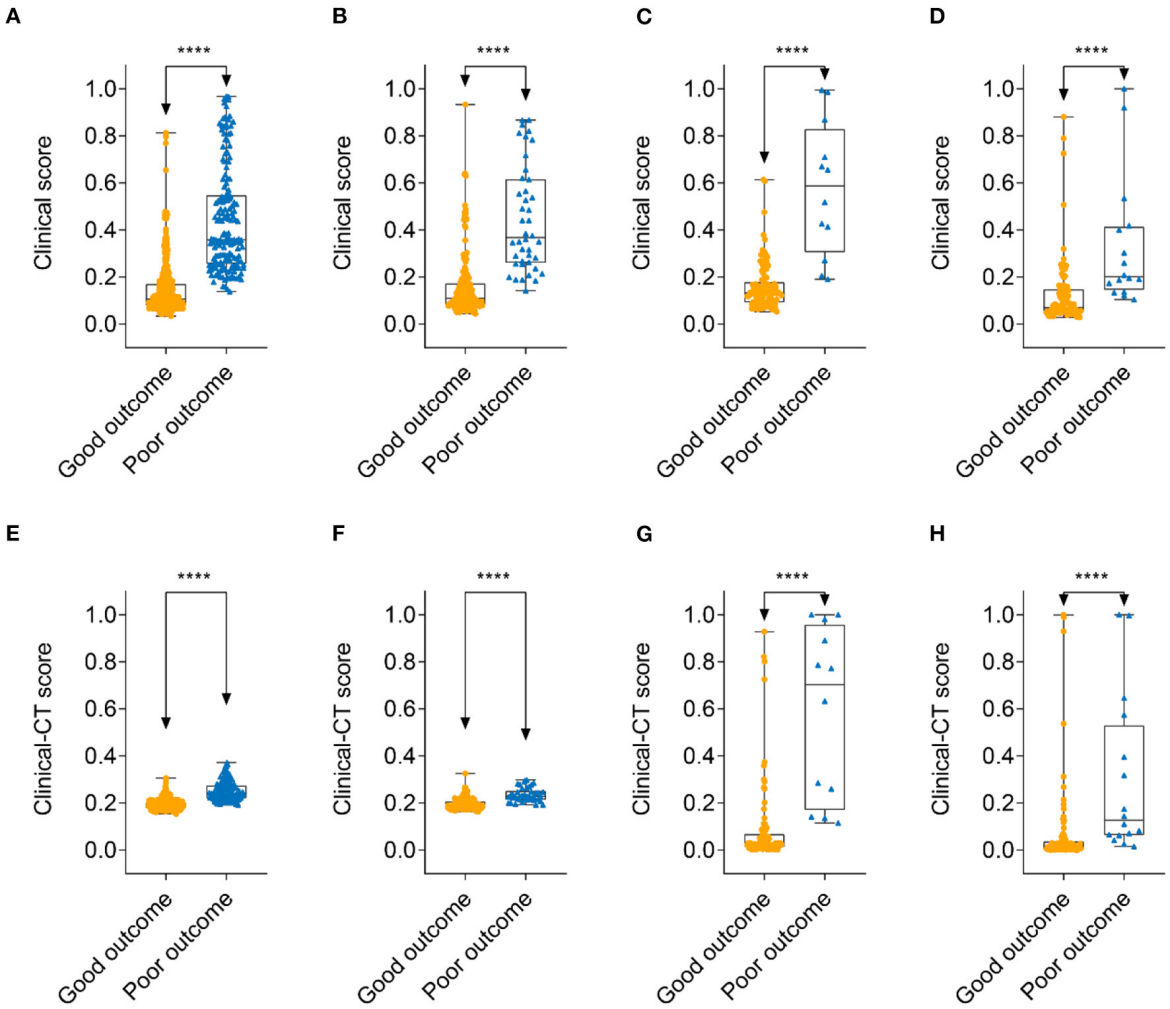
Comparison of clinical score and clinical-CT score between patients with 30-days good outcome and poor outcome. Clinical score: **(A)** training cohort; **(B)** internal validation cohort; **(C)** external validation cohort 1; and **(D)** external validation cohort 2. Clinical-CT score: **(E)** training cohort; **(F)** internal validation cohort; **(G)** external validation cohort 1; and **(H)** external validation cohort 2. ****denotes *p* < 0.0001.

A clinical nomogram was provided for the convenience of clinical score calculation (Figure 3A). Figure 3B shows the clinical-CT nomogram integrating the eight valuable clinical variables with the CT score. Akaike information criterion (AIC) of the two nomograms were −506.61 and −505.45, respectively. The clinical-CT nomogram yielded an AUC of 0.936 (95% CI: 0.917–0.951), sensitivity of 94.6%, specificity of 78.8%, accuracy of 81.8%, PPV of 52.0%, and NPV of 98.4% in the training cohort and AUC of 0.877 (95% CI: 0.825–0.918), sensitivity of 85.7%, specificity of 72.7%, accuracy of 75.2%, PPV of 43.4%, and NPV of 95.4% in the internal validation cohort. The clinical-CT nomogram achieved good performance in the external validation cohort 1 (AUC, 0.943, 95% CI: 0.882–0.979; sensitivity, 75.0%; specificity, 89.7%; accuracy, 88.1%; PPV, 47.4%; and NPV, 96.7%) and the external validation cohort 2 (AUC, 0.872, 95% CI: 0.796–0.928; sensitivity, 50.0%; specificity, 90.6%; accuracy, 83.9%; PPV, 43.8%; and NPV, 90.6%). However, AUCs of the clinical score and clinical-CT score were not significantly different in two external validation cohorts (*p* = 0.807 and 0.486) (Supplementary Figure 2). The cutoff value of clinical-CT score was 0.204, which could stratify patients into a low-risk or high-risk group. Figure 4 shows the comparison of clinical score and clinical-CT score between the patients with good and poor outcome, with significant differences in all the cohorts (all *p* < 0.0001). Supplementary Figure 3 shows the calibration curves of the clinical nomogram and clinical-CT nomogram.

### Clinical Usefulness of the CT Score, Clinical Nomogram, and Clinical-CT Nomogram

**Figure 5:**
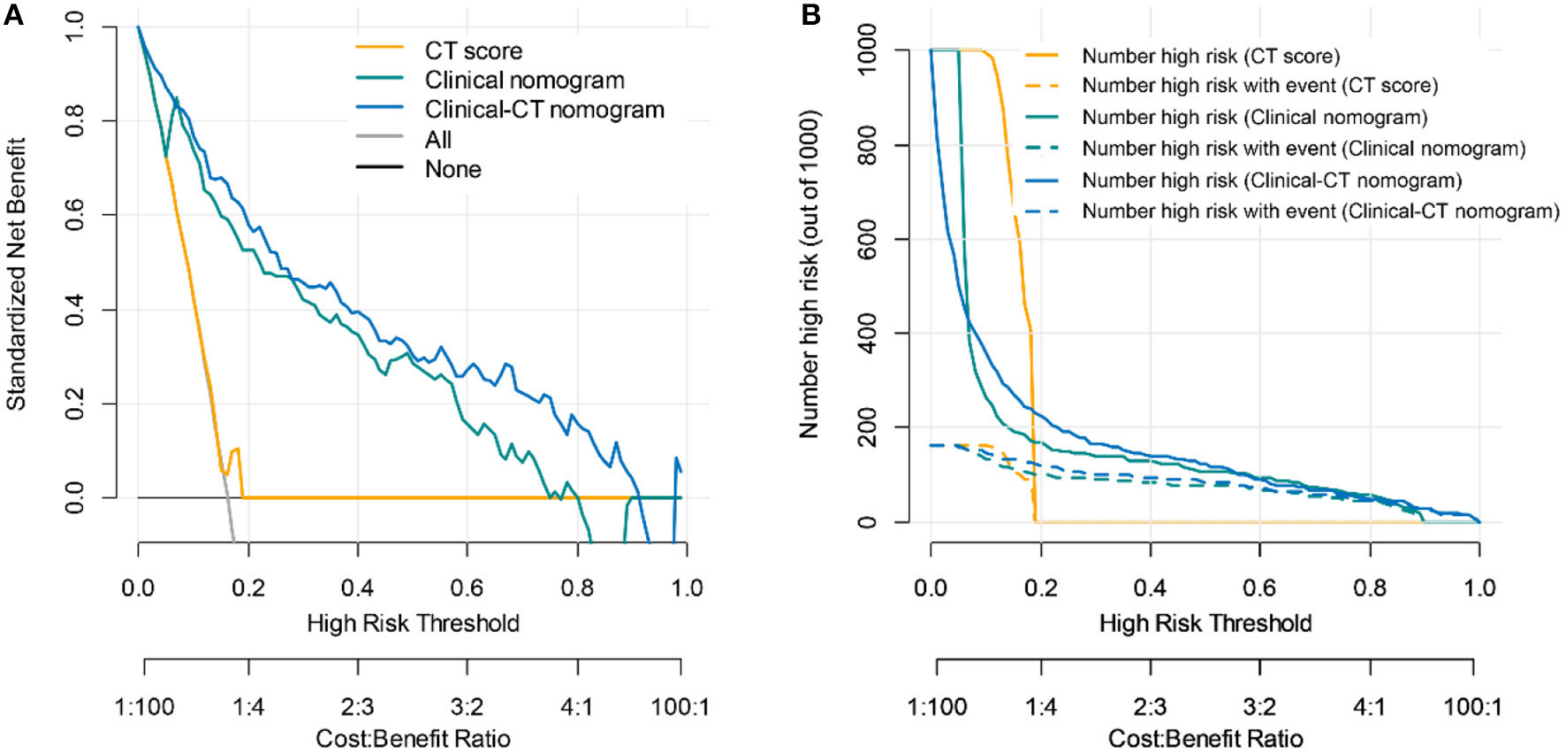
Clinical utility evaluation of the clinical score, CT nomogram, and clinical-CT nomogram for 30-days poor outcome prediction. **(A)** Decision curve analysis. Net benefit curves are plotted across risk or probability thresholds for an event (critical illness) for five options: “treat all” as if they are critically ill, “treat none” considering none is critically ill, treat according to critical illness by CT score, clinical nomogram, and clinical-CT nomogram. **(B)** Clinical impact curve of the CT score, clinical nomogram, and clinical-CT nomogram plotted the number of COVID-19 patients classified as high risk, and the number of cases classified high risk with severe COVID-19 at each high-risk threshold. The dotted yellow, green, and blue curves (number of high-risk individuals with outcome) denote the number of true positives at each threshold probability. The solid yellow, green, and blue lines (number of high-risk individuals) indicate the number of people who are classified as positive (high risk) by the CT score, clinical nomogram, and clinical-CT nomogram at each threshold probability.

The area under the decision curves shows the clinical utility of corresponding strategies. The clinical-CT nomogram (blue) showed more net benefit than that of clinical nomogram (green) or CT score (yellow), which were better than the “treat all” (gray) or “treat none” (black) strategies, indicating better clinical application of the clinical-CT nomogram. The decision curve (Figure 5A) and CIC (Figure 5B) showed that the clinical-CT nomogram had superior standardized net benefit and impact on the outcome of COVID-19 patients.

## Discussion

Due to the challenges that arise during the ongoing COVID-19 pandemic, we call for robust tools to aid in making complex clinical decisions. Clinical management of COVID-19 requires frequent monitoring and re-assessment of patients who may suffer from deterioration. Our models provide a reliable and accurate tool for risk quantification for 30-days poor outcome among COVID-19 patients during hospitalization. Our models exhibited relatively good discriminatory power, and external verification was also satisfactory. Of note, our models were applicable for guiding clinical decision-making.

Several elements of the clinical nomogram have been either established as prognostic markers or identified as risk factors for severe illness or death in patients with COVID-19. Elderly people are at higher risks for chronic diseases and more susceptible to COVID-19 infection (4). Older age is identified as a well-known risk factor for worse outcomes (e.g., respiratory failure and ICU admission) among patients with COVID-19 partially because age-related immune dysfunctions result from low-grade chronic inflammation (14). In addition, elderly patients were more likely to have underlying comorbidities, such as hypertension, diabetes, chronic lung disease, and cardiovascular disease, which complicated the treatment of COVID-19 and deteriorated the severity of disease.

Although the lung is the most affected organ by COVID-19, other organ dysfunction, including liver, kidney cardiac, and coagulation dysfunction, indicates poor survival outcomes (25). Although LDH is not a marker of a specific organ, the rise in LDH level indicates an increase in the activity and extent of lung injury, especially in critically ill patients with COVID-19 (26). AST elevation was common and may be due to cholangiocyte dysfunction and other causes, such as drug-induced and systemic inflammatory response-induced liver injuries (27). Liver injury was independently associated with the need for ICU admission, mechanical ventilation, and/or death in COVID-19 patients (27). Previous studies suggested a 3–11% incidence of acute kidney injury (AKI) in patients with COVID-19 (28). Around 9.6 and 13.7% of patients had elevated serum creatinine and blood urea nitrogen, respectively (28). The etiology of AKI in COVID-19 is thought to be multifactorial, and the mechanism of kidney involvement may include direct cellular injury due to the virus or sepsis leading to cytokine storm syndrome (29). AKI is closely associated with the severity and prognosis of COVID-19 patients (30). Coagulation dysfunction is more common in patients with severe and critically ill COVID-19. Elevation of PT and D-dimer indicated a hypercoagulable state in patients at the early stage, which was an independent predictor of requiring critical care support or in-hospital mortality (31, 32). The coagulation indicators such as D-dimer and PT should be monitored as early as possible in order to detect thrombotic complications. We strongly suggest that special care of multiple organ dysfunction should be included in the treatment of patients with COVID-19 during hospitalization.

While serum sodium has not yet been related to COVID-19, it has been independently and consistently associated with adverse outcomes in other populations (33) and disease states (34, 35). Previous studies showed that admission FBG was an independent predictor for poor prognosis of COVID-19 patients (36, 37). Hyperglycemia is mainly caused by pre-existing diabetes and stress-induced hyperglycemia. Diabetes has been identified as an important risk factor for mortality and progression in COVID-19 patients (38). In addition to pre-existing diabetes, elevation of FBG level at admission could also be due to stress hyperglycemia. Stress hyperglycemia is common in patients without diabetes, which is more concerning in clinical practice. Stress hyperglycemia may be induced by a decrease of both insulin secretion and the worsening of insulin resistance; it may produce organ damage by inducing endothelial dysfunction and thrombosis through the glycation process and oxidative stress generation (39). Glucose control helps prevent and control infections and their complications (40). Accordingly, well-controlled blood glucose may lead to improved outcomes of patients with COVID-19.

Chest CT plays an indispensable role in the detection, diagnosis, and follow-up of COVID-19 pneumonia. Visual CT score is a semi-quantitative marker that can assess the disease severity of COVID-19 according to lung involvement in the clinical setting (41). The index is simple, reproducible, and readily available in daily practice without image post-processing. However, it is usually visually calculated by radiologists, which is somewhat subjective with variability that unable to quantitatively assess the disease severity and is also time-consuming (42). Despite these limitations, some previous studies suggested that CT score was highly correlated with laboratory findings and disease severity, which could serve as a biomarker of predicting the outcome of COVID-19 patients (41, 43–51). However, our study might suggest that admission CT score is not a significant predictor for longer-term prognosis. Interestingly, although adding CT score to the clinical nomogram could not improve the predictive performance, the combination of both had more net benefit than clinical nomogram alone.

Based on the identified predictors, clinical nomogram was developed for doctors to quickly assess the risk with sample clinical and laboratory features and facilitated early decision making of COVID-19. Nomogram as the visualization of these models could serve as a simple tool for physicians and patients to calculate individual risk. Our model is able to stratify COVID-19 patients into low- and high-risk groups for developing 30-days poor outcome. The clinical usefulness of our models was tested, and the results showed that although the clinical nomogram and clinical-CT nomogram shared similar discrimination power, the latter had a better clinical application. Several previous studies also showed the clinical usefulness of prognostic models in the management of COVID-19 patients (52–60).

## Limitations

This study also has some potential limitations. First, we included the retrospective nature of the sample that may introduce potential risks of bias in the data particularly if this involved convenience sampling or potentially crucial predictors were not available. Second, the training and validation of the model are restricted to several populations in China; further validation using external populations would improve the generalizability of the model. Third, CT score is somewhat subjective with large intra- and inter-observer variability obtained from the initial CT examination; CT-based radiomics or deep learning and follow-up CT scan may provide more prognostic information. Fourth, indicators of cardiac injury such as creatine kinase and creatine kinase MB were not analyzed due to insufficient data. This may limit the model fit and introduce bias if the data were not missing at random. Fifth, the potential duration of infection prior to presentation need not be indicated, which may be useful to assess rate of progression of infection in patients independently of biomarkers used in this study. Sixth, this model was not applicable for patients with critical illness at admission, which may result in inclusion bias. Seventh, COVID-19 triaging might lead to less severe cases having delayed testing, thereby skewing the inclusion set to a more critical status; thus, the performance of model may be overestimated. Eighth, self-medication of patients before admission may affect the clinical and laboratory results, but it should have no major effect on the models as long as these medications were random. Finally, the identification of predictors depends on available features, feature selection method used, and sample size of studies.

## Conclusion

This study developed and externally validated a simple predictive model of 30-days poor outcome for hospitalized patients with COVID-19 based on objective data that are routinely used in clinical setting. Clinical nomogram integrated eight optimal predictors of 30-days poor outcome, including age, lactic dehydrogenase, aspartate aminotransferase, prothrombin time, D-dimer, serum creatinine, serum sodium, and fasting blood glucose. We found that older age, multiple organ dysfunction, hyponatremia, and hyperglycemia were key prognostic factors of COVID-19 patients. Although the addition of CT score to the clinical nomogram could not enhance its predictive performance, the combination of eight clinical predictors and CT score might be more clinically useful than clinical nomogram alone. Early detection of patients who are likely to develop poor outcome is of great importance, which may help select patients at risk of rapid deterioration who should require high-level monitoring and more aggressive treatment.

## Data Availability Statement

The original contributions presented in the study are included in the article/Supplementary Materials, further inquiries can be directed to the corresponding author/s.

## Ethics Statement

The studies involving human participants were reviewed and approved by The First Affiliated Hospital of Jinan University. The ethics committee waived the requirement of written informed consent for participation.

## Author Contributions

SZ, QZ, BZ, and QL designed the study. XZ, SL, and WC analyzed and interpreted the data. ML, ZC, LvC, and YD collected the data. JY, QC, and LuC supervised the data. BZ and QL wrote the original draft of the manuscript. All authors contributed to the final editing.

## Conflict of Interest

The authors declare that the research was conducted in the absence of any commercial or financial relationships that could be construed as a potential conflict of interest.
