# Supplementary material for: Clinical Utility of a Nomogram for Predicting 30-Days Poor Outcome in Hospitalized Patients With COVID-19: Multicenter External Validation and Decision Curve Analysis
Source: Front Med (Lausanne). 2020 Dec 23;7:590460. doi: 10.3389/fmed.2020.590460 (PMC7785751; doi:10.3389/fmed.2020.590460)
Supplement: Supplementary file 1 [file Data_Sheet_1.PDF]

Supplementary Materials

Supplementary document. CT acquisition and reconstruction parameters

Primary cohort: All patients underwent chest CT scans by a 64-slice CT scanner (Siemens Definition AS + 128, Forchheim, Germany). Each patient was scanned from the lung apex to the diaphragm during a breath-hold at end full inspiration and at end normal-expiration. To reduce breathing artifacts, patients were instructed on breath-holding. No contrast agent was administered. CT acquisition was executed as follows: tube voltage, 120 KV; tube current, auto mAs; pitch, 1.2; Rotation time, 0.5 s; field of view, 330 mm × 330 mm. Lung images were reconstructed at a slice thickness of 1.0-1.25 mm using I50 medium sharp algorithm. Lung window level and window width were set as -530-430 Hounsfield units (HU) and 1400-1600 HU, respectively.

External validation cohort 1: Patients underwent chest CT scans by CT 64 scanner (GE Medical System), Siemens Emotion 16 scanner (Siemens Healthineers; Erlangen, Germany), or ICT 128 scanner (Philips Healthcare, Netherlands). Each patient was scanned from the lung apex to the diaphragm during a breath-hold at end full inspiration and at end normal-expiration. To reduce breathing artifacts, patients were instructed on breath-holding. No contrast agent was administered. CT acquisition of GE 64 scanner was executed as follows: tube voltage, 120 KV; tube current, 260 mAs; pitch, 0.984; and slice thickness reconstructions of 0.625 mm. CT acquisition of Siemens 16 scanner was executed as follows: tube voltage, 130 KV; automatic tube current; pitch, 1.5; and slice thickness reconstructions of 1.0 or 0.6 mm. CT acquisition of ICT 128 scanner was executed as follows: tube voltage, 120 KV; automatic tube current; pitch, 0.7; collimation, 0.625 mm and slice thickness reconstructions of 1.0 or 0.67 mm.

External validation cohort 2: Chest CT scans were performed with two multi-detector CT scanners (GE Optima 520 Pro, America; Philips Brilliance iCT, Netherlands). Each patient was scanned from the lung apex to the diaphragm during a breath-hold at end full inspiration and at end normal-expiration. CT acquisition was executed as follows: tube voltage, 120kVp; tube current, 250mA; matrix size, 512 × 512; slice thickness, 1.25mm; slice spacing, 1.25 mm; field of view, 360 mm × 360 mm. No contrast agent was administered.

Supplementary Figure 1. Violin plots for the selected top-predictive variables consisted of (a) Age, (b) LDH, (c) AST, (d) PT, (e) Scr, (f) Na, (g) FBG, and (h) DD.

Note: * P<0.05, **P<0.01, ***P<0.001, ****P<0.0001.

Abbreviations: LDH, lactic dehydrogenase; AST, aspartate aminotransferase, PT, prothrombin time; Scr, serum creatinine; Na, serum sodium; FBG, fasting blood glucose, DD, D-dimer.

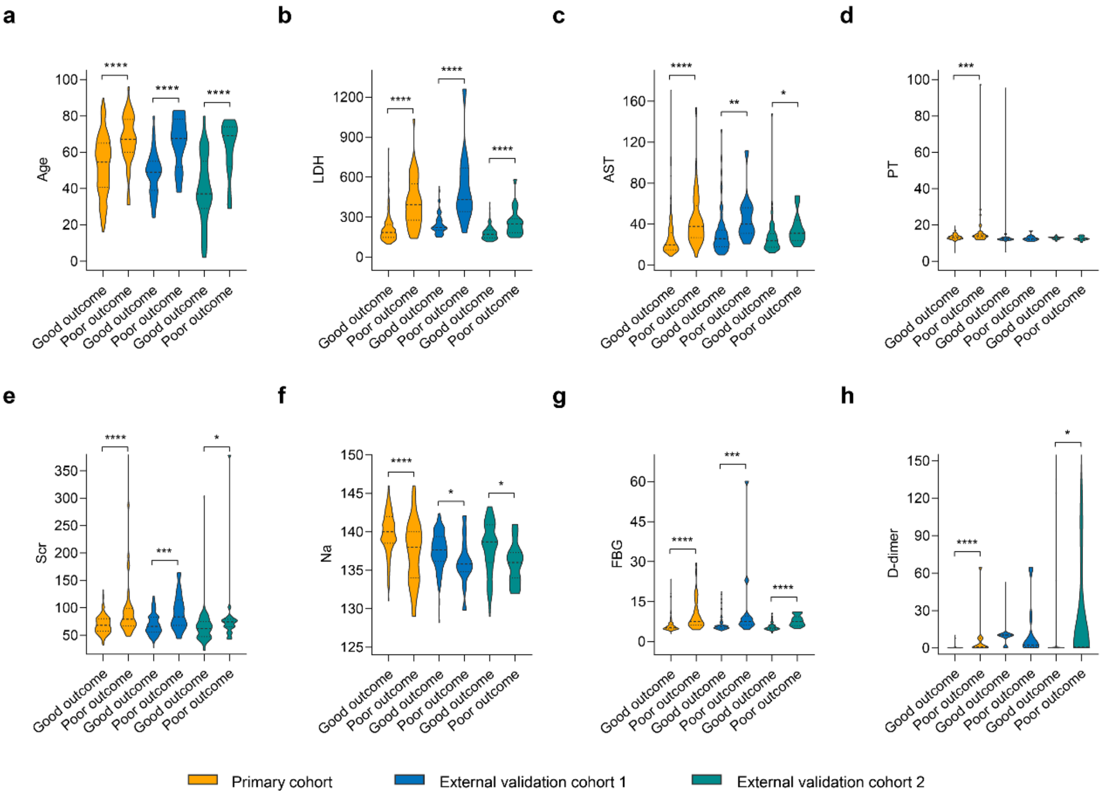

Supplementary Figure 2. Receiver-operator characteristic curves of the clinical nomogram and clinical-CT nomogram for 30-day poor outcome prediction: (a) training cohort; (b) internal validation cohort; (c) external validation cohort 1; (d) external validation cohort 2.

Abbreviation: CT, computed tomography.

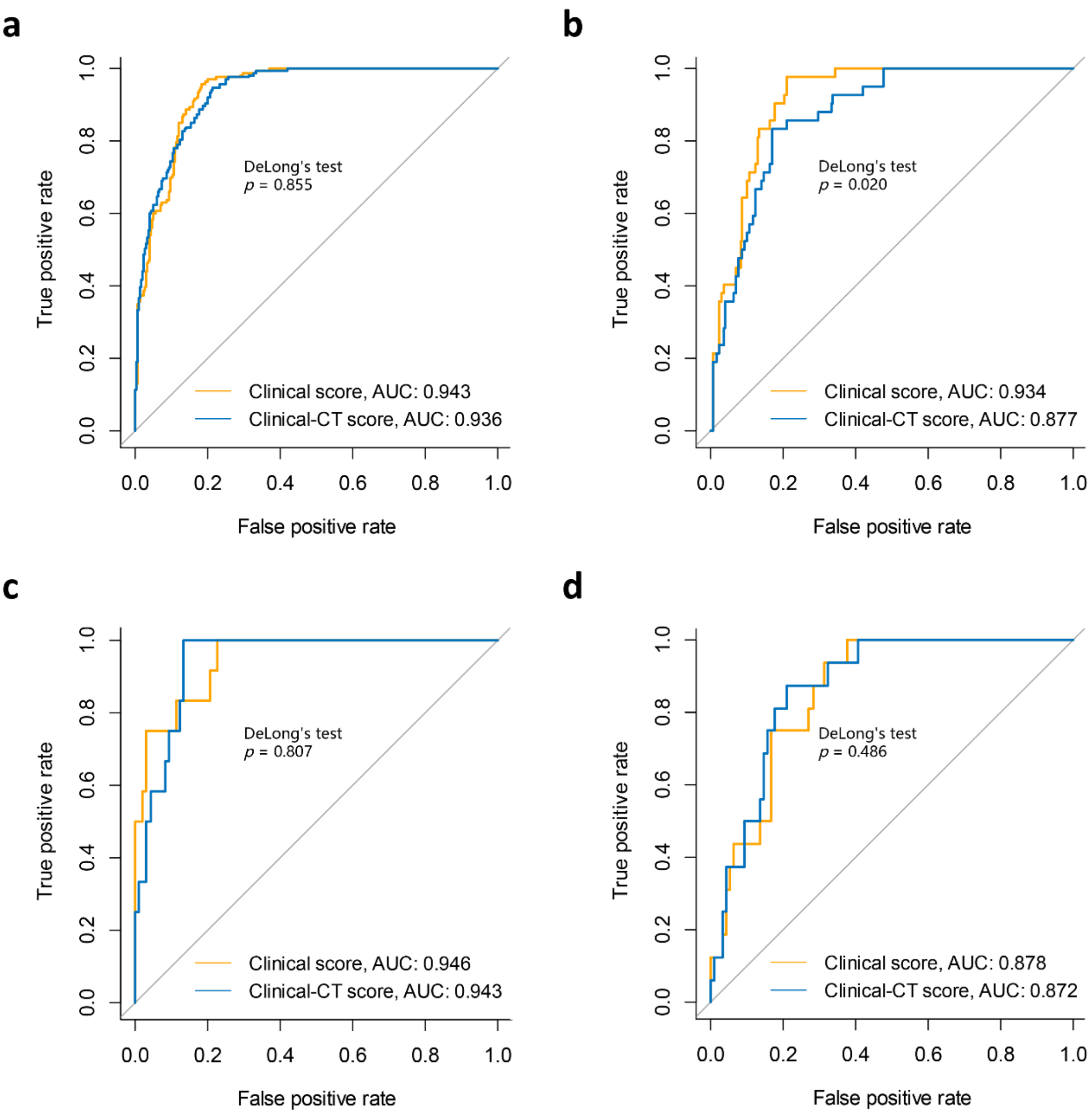

Supplementary Figure 3. Calibration curves of the clinical nomogram and clinical-CT nomogram for 30-day poor outcome prediction. Clinical nomogram: (a) internal validation cohort; (b) external validation cohort 1; and (c) external validation cohort 2. Clinical-CT nomogram: (d) internal validation cohort; (e) external validation cohort 1; and (f) external validation cohort 2.

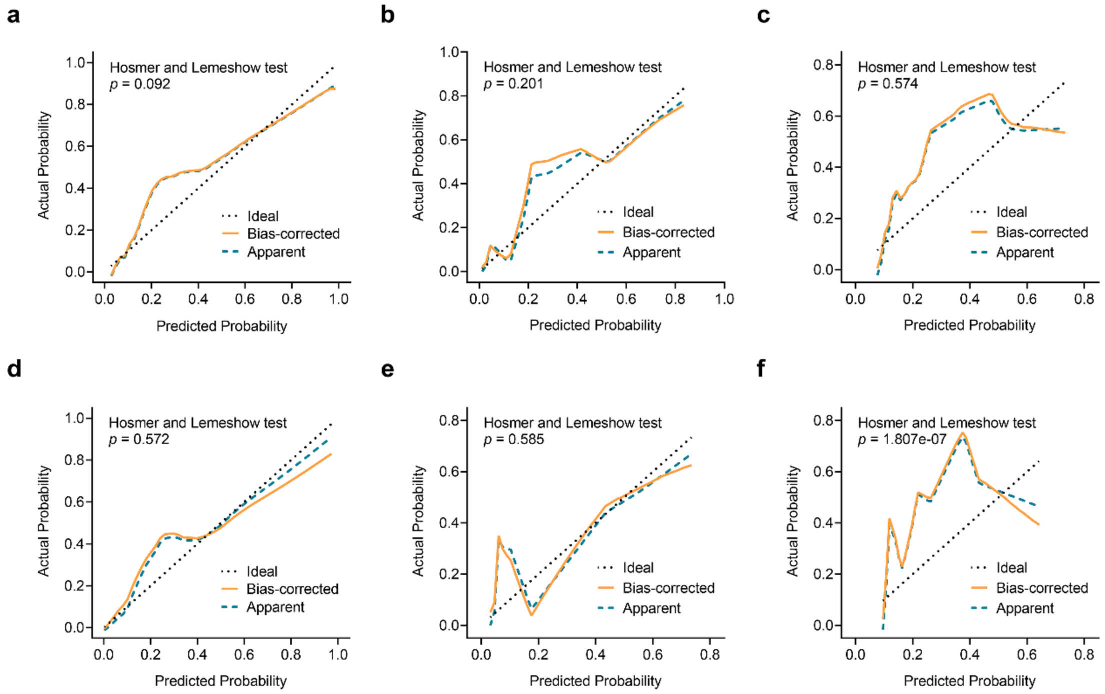

Supplementary Table 1. Clinical and laboratory characteristics of patients in two external validation cohorts

	External validation cohort 1				External validation cohort 2			
	Total (n = 110)	30-day poor outcome			Total (n = 118)	30-day poor outcome		
		Yes (n = 12)	No (n = 98)	P value		Yes (n = 19)	No (n = 99)	P value
**Age (years), median (IQR)**	50.0 (39.0, 58.0)	67.5 (51.8, 78.3)	49.0 (39.0, 55.0)	<0.001	40.5 (30.0, 62.0)	69.0 (52.0, 74.0)	37.0 (29.0, 55.0)	<0.001
**Sex, n (%)**								
male	69 (62.7)	8 (66.7)	61 (62.2)	1	66 (55.9)	13 (68.4)	53 (53.5)	0.231
female	41 (37.3)	4 (33.3)	37 (37.8)		52 (44.1)	6 (31.6)	46 (46.5)	
**Comorbidities, n (%)**								
Hypertension	11 (10.0)	3 (25.0)	8 (8.2)	0.099	22 (18.6)	9 (47.4)	13 (13.1)	0.002
Coronary heart disease	2 (1.8)	0	2 (2.0)	1	4 (3.3)	2 (10.5)	2 (2.0)	0.121
Diabetes	4 (3.6)	0	4 (4.1)	1	14 (11.9)	6 (31.6)	8 (8.1)	0.011
Hepatitis	1 (0.9)	0	1 (1.0)	1	8 (6.8)	3 (15.8)	5 (5.1)	0.117
Chronic lung disease	4 (3.6)	1 (8.3)	3 (3.1)	0.374	5 (4.2)	3 (15.8)	2 (2.0)	0.029
**Symptoms and signs, n (%)**								
Fever	92 (83.6)	11 (91.7)	81 (82.7)	0.686	77 (65.3)	14 (73.7)	63 (63.6)	0.4
Cough	60 (54.5)	9 (75.0)	51 (52.0)	0.132	51 (43.2)	8 (42.1)	43 (43.4)	0.915
Sputum	3 (2.7)	0	3 (3.1)	1	18 (15.3)	2 (10.5)	16 (16.2)	0.734
Rhinobyon	0	0	0	NA	5 (4.2)	0	5 (5.1)	1
Headache	0	0	0	NA	5 (4.2)	1 (5.3)	4 (4.0)	1
Sore throat	2 (1.8)	1 (8.3)	1 (1.0)	0.207	17 (14.4)	1 (5.3)	16 (16.2)	0.301
Fatigue	5 (4.5)	0	5 (5.1)	1	11 (9.3)	5 (26.3)	6 (6.1)	0.016
Myalgia	0	0	0	NA	8 (6.8)	1 (5.3)	7 (7.1)	1
Chest pain/Chest distress	4 (3.6)	0	4 (4.1)	1	5 (4.2)	1 (5.3)	4 (4.0)	1
Shortness of breath	5 (4.5)	1 (8.3)	4 (4.1)	0.445	3 (2.5)	0	3 (3.0)	1
Diarrhea	1 (0.9)	0	1 (1.0)	1	5 (4.2)	1 (5.3)	4 (4.0)	1
Chills	3 (2.7)	1 (8.3)	2 (2.0)	0.295	8 (6.8)	2 (10.5)	6 (6.1)	0.613
Asymptomatic	0	0	0	NA	13 (11.0)	2 (10.5)	11 (11.1)	1
**Laboratory findings, median (IQR)**								
WBC (× 10^9/L)	4.9 (4.0, 6.3)	6.8 (5.0, 13.7)	4.9 (4.0, 6.0)	<0.001	5.0 (4.0, 6.2)	5.2 (3.1 6.9)	5.0 (4.0, 6.1)	0.733
Neutrophil (× 10^9/L)	3.1 (2.3, 4.5)	5.7 (2.8, 12.1)	3.0 (2.3, 4.1)	0.012	3.0 (2.3, 4.6)	3.8 (2.0, 4.6)	3.0 (2.4, 4.4)	0.578
Lymphocyte (× 10^9/L)	1.2 (0.9, 1.5)	0.9 (0.5, 1.4)	1.2 (1.0, 1.5)	0.055	1.1 (0.8, 1.7)	1.0 (0.6, 1.1)	1.2 (0.9, 1.8)	0.006
NLR	2.6 (1.6, 4.5)	7.8 (2.2, 14.1)	2.4 (1.6, 4.0)	0.005	2.7 (1.7, 4.4)	3.6 (2.4, 6.9)	2.5 (1.6, 4.1)	0.026
LDH (U/L)	228.5 (204.3, 307.8)	431.0 (342.0, 669.5)	222.0 (201.8, 276.3)	<0.001	177.5 (147.5, 218.0)	249.0 (181.0, 289.0)	172.0 (143.0, 195.0)	<0.001
Hemoglobin (g/L)	132.0 (118.0, 144.0)	137.0 (11.5, 144.0)	132.0 (118.0, 143.3)	0.818	134.0 (121.0, 145.0)	132.0 (121.0, 148.0)	134.0 (122.0, 144.0)	0.901
Platelet (g/L)	199.5 (147.0, 261.3)	197.0 (133.5, 260.3)	199.5 (150.0, 261.3)	0.673	210.5 (159.3, 251.0)	172.0 (121.0, 198.0)	212.7 (166.0, 255.0)	0.003
Albumin (g/L)	36.8 (34.0, 39.8)	34.9 (30.1, 36.5)	37.6 (34.3, 40.3)	0.007	39.0 (36.9, 42.7)	35.9 (33.1, 38.2)	39.8 (38.4, 43.3)	<0.001
AST (U/L)	27.2 (19.3, 37.5)	40.1 (31.3, 56.0)	26.0 (18.2, 34.4)	0.002	25.0 (18.0, 33.0)	31.3 (24.0, 44.0)	23.8 (17.7, 31.0)	0.002
ALT (U/L)	23.0 (15.3, 39.4)	28.7 (16.8, 43.3)	22.7 (15.0, 37.6)	0.412	16.7 (11.8, 26.6)	21.5 (14.0, 36.9)	16.1 (11.7, 25.2)	0.045
DBIL (μmol/L)	4.5 (3.4, 6.0)	5.8 (4.3, 6.6)	4.3 (3.3, 5.8)	0.074	4.2 (3.0, 4.9)	5.3 (3.0, 10.0)	4.2 (3.0, 4.5)	0.02
IBIL (μmol/L)	7.0 (5.6, 10.4)	7.1 (6.5, 8.9)	7.0 (5.5, 10.7)	0.996	7.5 (4.8, 8.4)	6.0 (5.3, 11.0)	8.0 (4.2, 8.4)	0.674
TBIL (μmol/L)	12.1 (9.1, 16.8)	12.8 (11.1, 15.5)	11.7 (9.1, 17.2)	0.562	12.6 (7.7, 13.2)	11.9 (8.5, 20.6)	12.6 (7.6, 12.6)	0.358
APTT (s)	29.8 (27.0, 34.1)	30.2 (28.2, 34.1)	29.7 (26.3, 34.1)	0.625	35.8 (34.1, 40.6)	35.2 (31.1, 40.5)	36.7 (34.1, 40.7)	0.417
PT (s)	12.3 (11.8, 12.7)	12.3 (11.7, 13.6)	12.3 (11.8, 12.7)	0.740	12.7 (12.2, 13.2)	12.4 (11.9, 13.6)	12.9 (12.3, 13.2)	0.285
D-dimer (μg/ml)	10.4 (2.6, 10.4)	2.5 (0.8, 20.4)	10.4 (10.4, 10.4)	0.121	0.4 (0.2, 0.9)	0.7 (0.4, 98.0)	0.3 (0.2, 0.6)	0.001
Creatinine (μmol/L)	68.1 (56.5, 83.4)	83.5 (67.7, 114.1)	65.8 (55.4, 82.9)	0.017	65.0 (48.0, 77.0)	74.0 (65.0, 79.0)	62.0 (47.0, 75.0)	0.055
hs-CRP (mg/L)	24.5 (5.1, 65.6)	86.5 (48.5, 146.2)	17.5 (4.7, 54.8)	<0.001	5.0 (2.6, 8.3)	15.2 (5.0, 39.0)	5.0 (2.3, 5.0)	0.004
Procalcitonin (ng/ml)	0.2 (0.1, 3.8)	0.7 (0.2, 5.9)	0.2 (0.1, 3.8)	0.282	0.2 (0.1, 0.2)	0.1 (0.1, 0.2)	0.2 (0.1, 0.2)	0.767
Potassium (mmol/L)	4.1 (3.8, 4.5)	3.8 (3.4, 4.1)	4.2 (3.9, 4.5)	0.013	3.8 (3.5, 4.1)	3.6 (3.5, 3.9)	3.9 (3.6, 4.1)	0.059
Sodium (mmol/L)	137.5 (136.0, 139.3)	135.8 (134.8, 137.8)	137.7 (136.2, 139.4)	0.037	138.0 (135.1, 140.1)	136.0 (134.0, 137.3)	138.7 (136.0, 140.9)	0.009
Chloride (mmol/L)	101.4 (99.6, 103.6)	7.6 (97.3, 101.3)	101.8 (99.8, 103.8)	0.009	100.1 (96.9, 103.1)	98.7 (93.7, 101.0)	100.1 (97.5, 103.1)	0.023
FBG (mmol/L)	5.6 (5.0, 6.7)	7.6 (6.3, 9.6)	5.5 (5.0, 6.0)	0.001	5.4 (4.8, 6.3)	7.5 (6.0, 9.3)	5.1 (4.7, 6.0)	<0.001

Note: Data were median (interquartile range, IQR) or number (percentage). P values were calculated by Mann-Whitney U test, χ^2 test or Fisher's exact test, as appropriate. Abbreviations: WBC, white blood cells; LDH, lactate dehydrogenase; AST, aspartate aminotransferase; ALT, alanine aminotransferase; TBIL, Total Bilirubin; DBIL, Direct Bilirubin; IBIL, indirect bilirubin; APTT, activated partial thromboplastin time; PT, prothrombin time; hs-CRP, high-sensitivity C-reactive protein; NLR, neutrophil-lymphocyte ratio; FBG, fasting blood glucose.
